# Influence of Tooth Morphology on Local Mesh Density Distribution in Intraoral Scanner-Derived STL Models of Selected Maxillary Teeth

**DOI:** 10.3390/dj14050252

**Published:** 2026-04-27

**Authors:** Dubravka Knezović Zlatarić, Maja Žagar, Egon Neskusil, Daren Dreo Bračun, Robert Ćelić

**Affiliations:** 1 Department of Removable Prosthodontics, School of Dental Medicine, University of Zagreb, 10000 Zagreb, Croatia; mpavic@sfzg.hr (M.Ž.); celic@sfzg.hr (R.Ć.); 2 Clinical Hospital Center Zagreb, 10000 Zagreb, Croatia; 3 School of Dental Medicine, University of Zagreb, 10000 Zagreb, Croatia; egon.neskusil@gmail.com (E.N.); daren.dreobracun@gmail.com (D.D.B.)

**Keywords:** intraoral scanner, STL mesh, mesh density distribution, tooth morphology, digital surface reconstruction, maxillary teeth, digital dentistry

## Abstract

**Background/Objectives:** The quality of intraoral scanner-derived digital models depends not only on deviation-based accuracy, but also on how scanned surfaces are reconstructed into a polygonal mesh. The aim of this prospective within-subject observational study was to evaluate whether tooth morphology influences local mesh density distribution in intraoral scanner-derived STL models of selected maxillary teeth. **Methods:** Twenty participants underwent maxillary intraoral scanning using a Medit i900 wired intraoral scanner under standardized clinical conditions. For each participant, the buccal surfaces of the maxillary right central incisor (11), canine (13), first premolar (15), and first molar (16) were selected as regions of interest. Surface area (A), number of vertices (V), and number of faces (F) were recorded, and the surface-normalized mesh density parameters vertices per unit area (V/A) and faces per unit area (F/A) were calculated. Comparisons among tooth types were performed using repeated-measures analysis of variance (ANOVA) with Bonferroni post hoc correction. **Results:** Significant differences were found among tooth types for both V/A and F/A (*p* < 0.001). Mean V/A values were 18.2 ± 1.9 for tooth 11, 19.8 ± 1.4 for tooth 13, 23.8 ± 1.7 for tooth 15, and 22.9 ± 2.0 vertices/mm^2^ for tooth 16. Mean F/A values were 34.3 ± 3.6, 37.5 ± 2.7, 44.4 ± 3.3, and 42.9 ± 3.8 faces/mm^2^, respectively. Post hoc comparisons showed significant differences between teeth 11 and 13, 11 and 15, 11 and 16, 13 and 15, and 13 and 16, whereas no significant difference was observed between teeth 15 and 16. **Conclusions:** Tooth morphology significantly influenced local mesh density distribution in intraoral scanner-derived STL models of selected maxillary teeth. These findings suggest that local anatomical form affects STL mesh reconstruction under standardized in vivo scanning conditions and support local mesh density analysis as a useful complementary approach to conventional deviation-based digital assessment.

## 1. Introduction

The integration of intraoral scanners (IOSs) into contemporary dental workflows has substantially changed the acquisition of dental impressions by enabling direct digital capture of oral structures and facilitating CAD/CAM-based treatment planning and manufacturing. Current evidence indicates that IOS systems can provide clinically useful digital impressions, particularly for limited-span applications, although their accuracy remains influenced by multiple technical and procedural variables, including operator-related and patient-related factors [1,2,3].

The accuracy of IOS-derived datasets is commonly described in terms of trueness and precision, most often assessed by the superimposition of standard tessellation language (STL) files and the calculation of three-dimensional deviations. Previous studies have shown that complete-arch digital impressions are susceptible to cumulative deviation and that scanner performance may vary according to the extent of the scanned area and the evaluation method used [4,5,6].

In addition to global accuracy outcomes, the quality of a digital model also depends on how the scanned surface is reconstructed as a polygonal mesh. In STL files, surface geometry is represented by a network of triangles, and this triangulated structure may vary according to scanner hardware, software version, scan size, and surface characteristics of the scanned object. Recent reviews have emphasized that IOS accuracy is affected not only by the scanner itself, but also by software-related and operator-related factors, including scanning distance, scanning strategy, ambient conditions, extension of the scan, and patient-specific intraoral conditions [1,2,3,6,7,8,9].

Surface morphology appears to be particularly relevant to digital data quality. Asar et al. demonstrated a strong relationship between the surface topography of prepared tooth surfaces and the data quality of IOS-derived digital impressions, and their mesh-based analysis showed significant differences in the number of triangulation points across files of different resolutions [10]. In addition, studies assessing the effect of tooth type on scan accuracy suggest that local anatomy may influence the way dental surfaces are captured and reconstructed [3,11].

Tooth type may therefore influence digital acquisition and surface reconstruction. Son and Lee reported that the accuracy of dental 3D scans differed significantly according to tooth type, with intraoral scanner accuracy tending to worsen from anterior to posterior regions [11]. Similarly, complete-arch and large-area scans have repeatedly shown that increased anatomical complexity and scan extent are associated with greater reconstruction challenges, supporting the relevance of localized analyses at the tooth level [4,5,6,12,13].

The aim of this prospective within-subject observational study was to evaluate whether tooth morphology influences local mesh density distribution in intraoral scanner-derived STL models by comparing different tooth types within the same individuals. The null hypothesis was that tooth type would not significantly affect the surface-normalized mesh density parameters, expressed as vertices per unit area (V/A) and faces per unit area (F/A).

## 2. Materials and Methods

### 2.1. Participants

A total of 20 participants were recruited from among second- to fifth-year students at the School of Dental Medicine, University of Zagreb. The sample included 12 women (mean age 22.5 ± 1.7 years) and 8 men (mean age 22.7 ± 2.6 years). All participants were informed about the aim and procedures of the study before enrollment and provided written informed consent. The study protocol was approved by the Ethics Committee of the School of Dental Medicine, University of Zagreb (approval no. 003-01/26-05/02; approved on 11 February 2026).

### 2.2. Eligibility Criteria

**Figure 1:**
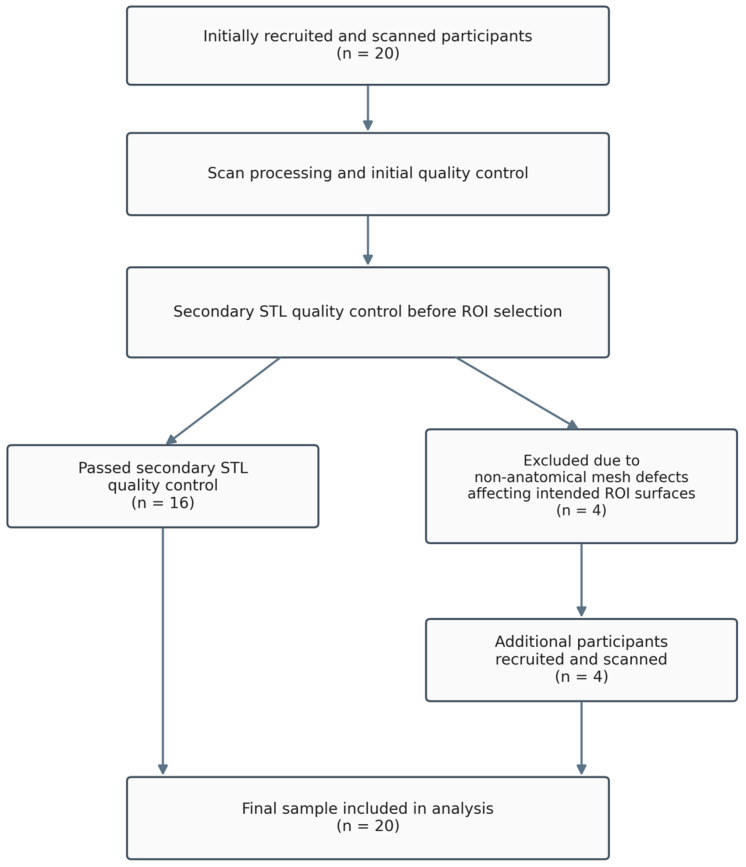
Flowchart of participant inclusion, exclusion, replacement, and final analysis.

Eligibility was determined according to predefined inclusion and exclusion criteria (Table 1). Only dentate participants with clinically healthy teeth and gingival tissues in the regions of interest were included (Figure 1).

### 2.3. Intraoral Scanning Procedure

All intraoral scans were obtained using a Medit i900 wired intraoral scanner (Medit Corp., Seoul, Republic of Korea) equipped with the large scanner tip and operated using Medit Link version 3.4.9 and Medit Scan for Clinics version 1.13.9. The same software environment, case set-up and acquisition workflow were used for all participants, and no tooth-specific preparation or restoration-oriented scan mode was activated. In each participant, the maxillary dental arch was scanned under dry intraoral conditions by the same operator, who had approximately 6 months of daily clinical experience with the same scanner before the start of the study. A standardized scanning path was used in all cases, starting from the occlusal surfaces, followed by the palatal surfaces, and ending with the buccal surfaces. Scanning was performed continuously, at a consistent speed, without pauses, without returning to previously scanned areas, and without deletion or locking of scan segments, to maintain procedural consistency. The mean scanning time was approximately 80 s per scan, and the number of acquired frames was consistently approximately 4500 per scan.

### 2.4. Scan Processing and Quality Control

After acquisition, each maxillary scan was first reviewed by the scanning operator to assess overall scan integrity. Teeth 11, 13, 15 and 16 had to be clearly captured and free of non-anatomical defects before any post-processing was accepted. Based on this initial review, all scans were considered acceptable for further processing. The same standard scan-processing workflow was then applied to all scans, including the automatic hole-closing option. Automatic hole closing was permitted only when any mesh discontinuities were located outside teeth 11, 13, 15 and 16. All ROI STL files were exported using the same software workflow, without intentional modification of mesh resolution settings. Before ROI selection, each STL file was reviewed again by the examiner responsible for ROI selection. During this secondary quality control, four scans were found to show non-anatomical mesh defects affecting the intended ROI surfaces, which therefore did not appear smooth and continuous, and were excluded according to predefined exclusion criteria. These participants were replaced by four newly recruited and scanned participants to maintain the final sample size of 20 (Figure 1). The replacement scans were subjected to the same scanning, processing, and quality-control protocol before inclusion in the study. No manual editing or additional mesh modification was performed prior to ROI selection.

### 2.5. Selection of Regions of Interest and STL Export

**Figure 2:**
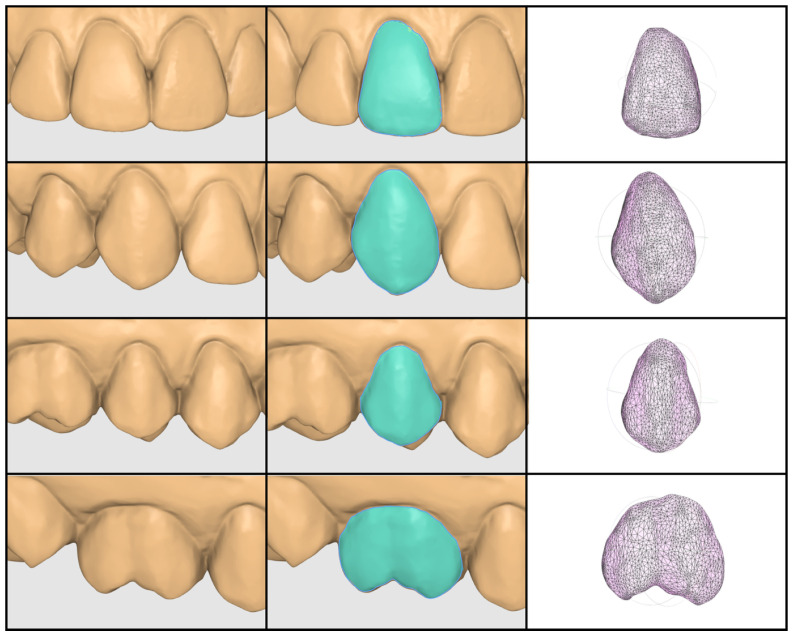
Representative STL model of the investigated tooth surfaces, ROI selection, and resulting mesh used for quantitative analysis.

For each participant, the buccal surfaces of the maxillary right central incisor (11), canine (13), first premolar (15) and first molar (16) were selected for analysis. ROI selection was performed for all samples by the same examiner using Medit Design 2.1.4 (Medit Corp., Seoul, Republic of Korea) and the largest available brush size (Figure 2). Before the start of the study, the examiner underwent 4 weeks of training in standardized ROI selection using the same software and protocol. Only the clinically visible buccal surface was included. The selection was confined within clearly defined anatomical boundaries, namely the gingival margin, the incisal edge or cusp tip, and the mesial and distal proximal line angles. Adjacent surfaces and non-buccal regions were intentionally excluded. After selection, each ROI was isolated as a separate object and exported as a separate STL file for subsequent mesh analysis.

### 2.6. Mesh Analysis

All exported ROI STL files were analyzed in MeshLab (2025.07; Visual Computing Lab, ISTI-CNR, Pisa, Italy) by an independent third examiner. For each ROI, the following parameters were recorded directly from the software: surface area (A, mm^2^), number of vertices (V), and number of faces (F) (Figure 2). The surface-normalized mesh density parameters, vertices per unit area (V/A) and faces per unit area (F/A), were calculated subsequently. No mesh cleaning, smoothing, remeshing, or other additional mesh modification procedures were performed.

### 2.7. Preliminary Scan Repeatability Assessment

Before the main study, preliminary repeatability testing of the scanning procedure was performed on two participants. Each participant was scanned four times by the same scanning operator using the same intraoral scanner and standardized scanning protocol: at baseline, after 10 min, after 1 h, and after 24 h. Repeated scans were compared with the baseline scan, and intra-operator scan repeatability was evaluated using root mean square (RMS) deviation analysis after superimposition. The mean RMS deviation across the repeated scans was 0.077 ± 0.012 mm (range: 0.061–0.095 mm), indicating good preliminary intra-operator repeatability of the standardized scanning protocol.

### 2.8. Preliminary ROI Selection Repeatability Assessment

After the training phase, a preliminary repeatability assessment was performed to evaluate examiner-dependent variability in ROI selection. The assessment included STL files of tooth 11 from five additional participants, independent of those used for scan repeatability testing. Two examiners performed ROI selection on the same tooth surfaces using Medit Design 2.1.4 (Medit Corp., Seoul, Republic of Korea), the same brush setting, and the same predefined anatomical boundaries. Repeatability was assessed using the study outcome variables, namely surface area, vertices per unit area (V/A), and faces per unit area (F/A), to evaluate examiner-dependent variability specifically related to ROI selection. Lower repeated-measurement variability was observed for one examiner, with mean absolute percentage differences of approximately 1.16% for surface area, 1.58% for V/A, and 1.42% for F/A, compared with 4.19%, 4.43%, and 4.62%, respectively, for the other examiner. The examiner with lower variability was therefore selected to perform ROI delineation for the main study sample.

### 2.9. Statistical Analysis

Statistical analysis was performed using IBM SPSS Statistics for Windows, version 29.0 (IBM Corp., Armonk, NY, USA). Descriptive statistics were calculated for all study variables. Surface area (A), number of vertices (V), and number of faces (F) were recorded for each ROI, and the derived mesh density parameters vertices per unit area (V/A) and faces per unit area (F/A) were calculated subsequently.

The main analysis was performed at the participant level by comparing the tooth types investigated within the same individuals. Since each participant contributed measurements for teeth 11, 13, 15, and 16, the data were analyzed using repeated-measures procedures. The distribution of the main outcome variables was assessed using the Shapiro–Wilk test. As no significant deviation from normality was observed, comparisons among tooth types were performed using repeated-measures analysis of variance (ANOVA). When the overall test was significant, pairwise post hoc comparisons were performed using Bonferroni correction.

The level of statistical significance was set at *p* < 0.05. No formal a priori sample size calculation was performed because the study was designed as an exploratory within-subject investigation of local mesh density parameters, and no directly applicable prior data were available for these outcome measures at the tooth level under the same in vivo conditions. The final sample size was determined on a feasibility basis, while maintaining a standardized study design and a homogeneous participant group to support internal consistency.

## 3. Results

A total of 20 participants contributed measurements for four tooth types (11, 13, 15, and 16). Before inferential analysis, the distribution of the main outcome variables was assessed using the Shapiro–Wilk test. No statistically significant deviation from normality was observed for either V/A or F/A across the investigated tooth types (all *p* > 0.05). Accordingly, comparisons among tooth types were performed using repeated-measures analysis of variance (ANOVA).

**Figure 3:**
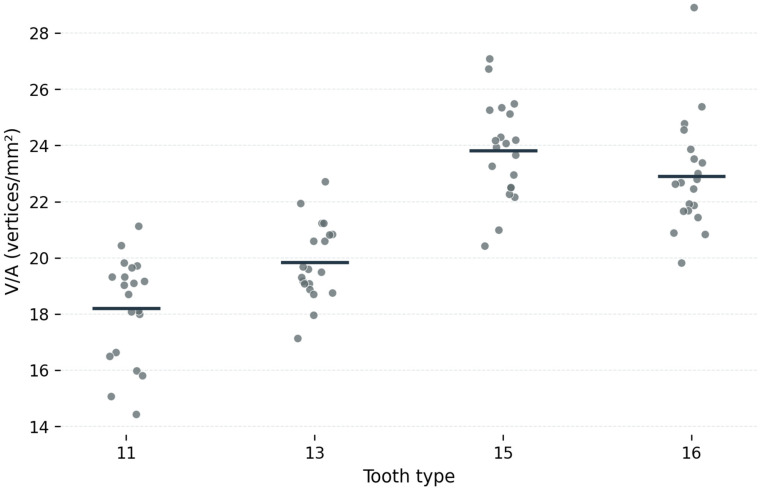
Average V/A profile across the investigated tooth types.

**Figure 4:**
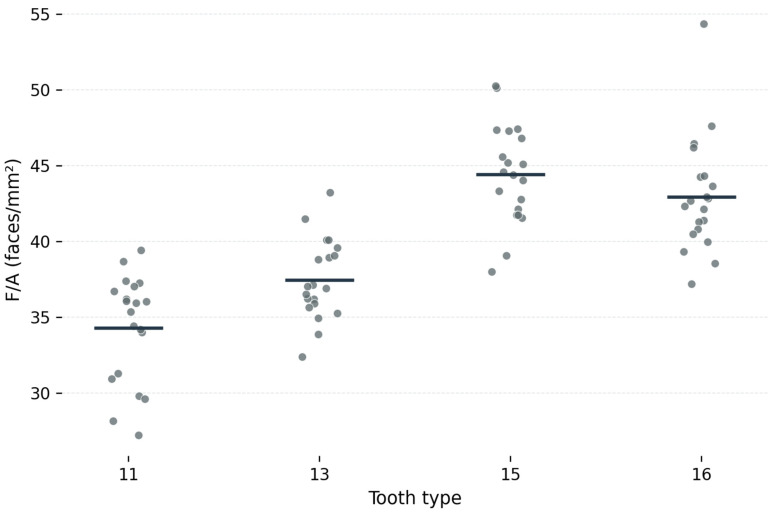
Average F/A profile across the investigated tooth types.

The surface-normalized mesh density parameters are presented in Table 2. Mean V/A values increased from tooth 11 to tooth 13, with the highest values observed for teeth 15 and 16 (Figure 3). Specifically, mean V/A values were 18.2 ± 1.9 vertices/mm^2^ for tooth 11, 19.8 ± 1.4 vertices/mm^2^ for tooth 13, 23.8 ± 1.7 vertices/mm^2^ for tooth 15, and 22.9 ± 2.0 vertices/mm^2^ for tooth 16. Mean F/A values followed the same pattern, with the lowest values for tooth 11, intermediate values for tooth 13, and the highest values for the posterior teeth (Figure 4). The corresponding F/A values were 34.3 ± 3.6 faces/mm^2^ for tooth 11, 37.5 ± 2.7 faces/mm^2^ for tooth 13, 44.4 ± 3.3 faces/mm^2^ for tooth 15, and 42.9 ± 3.8 faces/mm^2^ for tooth 16. Mean ROI surface area also differed among tooth types, with the largest mean area recorded for tooth 11 (70.3 ± 9.2 mm^2^) and the smallest for tooth 15 (35.6 ± 5.0 mm^2^), supporting the use of surface-normalized mesh density parameters for the primary analysis.

Repeated-measures ANOVA showed that tooth type had a statistically significant effect on both primary outcome variables. For V/A, the overall effect of tooth type was significant (F = 62.36, *p* < 0.001; Table 2). Likewise, for F/A, a significant overall effect was also observed (F = 54.79, *p* < 0.001; Table 2). These results indicate significant differences in local mesh density distribution among the investigated tooth types.

Post hoc pairwise comparisons with Bonferroni correction demonstrated significant differences between teeth 11 and 13, 11 and 15, 11 and 16, 13 and 15, and 13 and 16 for both V/A and F/A. In contrast, no significant difference was found between teeth 15 and 16 for either V/A or F/A.

## 4. Discussion

The null hypothesis was rejected because significant differences in the surface-normalized mesh density parameters V/A and F/A were found among the investigated tooth types. In the present study, tooth 11 showed the lowest mesh density values, tooth 13 showed intermediate values, and teeth 15 and 16 showed the highest values, with no statistically significant difference between the two posterior teeth. This pattern suggests that tooth morphology influenced local mesh density distribution in intraoral scanner-derived STL models under the standardized clinical conditions applied in the present study.

A key interpretative point is that V/A and F/A were not used as direct measures of trueness or precision, but as descriptors of local mesh density distribution relative to surface area. Accordingly, the present study was not designed as a conventional deviation-based accuracy study. Instead, it examined whether different tooth morphologies were associated with differences in local mesh density distribution under standardized in vivo scanning conditions. This distinction is important because, in an in vivo setting, obtaining a true geometric reference for each natural tooth surface is inherently difficult. The recent literature also suggests that mesh-related variables should not be interpreted as direct surrogates for conventional deviation-based measures of trueness [13]. The present findings should therefore be interpreted within the specific framework of local digital surface representation rather than as direct evidence of superior or inferior scanning accuracy.

The observed pattern is also anatomically plausible. The maxillary central incisor generally presents a broader and smoother buccal surface, whereas the canine shows a more pronounced transition in contour and greater convexity. The premolar and molar regions display more complex morphology and local curvature, which could affect how optical data are acquired and how the surface is reconstructed into a mesh. In this context, the progressive increase in V/A and F/A from tooth 11 to the posterior teeth appears biologically and technically coherent. This interpretation is consistent with the work of Son and Lee, who reported significant differences in scanner accuracy according to tooth type and showed that intraoral scanner performance tended to deteriorate from anterior toward posterior regions. Although the present study did not evaluate trueness directly, it similarly supports the broader principle that tooth type affects digital scan behavior [11].

The present findings are also supported by previous evidence linking surface topography to digital data quality. Asar et al. demonstrated strong correlations between the surface topography of prepared tooth surfaces and the data quality of IOS-derived digital impressions. Their mesh-based analysis further showed significant differences in triangulation points across STL files of different resolutions. Although their study investigated prepared teeth rather than intact buccal surfaces, the underlying concept is directly relevant to the present work: more complex or differently configured surfaces may be reconstructed differently at the digital mesh level. In that sense, the present study extends this concept to intact tooth surfaces and localized in vivo analysis [10]. However, previous studies have primarily focused on prepared teeth, export settings, mesh reduction, or global accuracy outcomes, whereas the present study specifically examined localized tooth-level mesh density distribution on intact buccal surfaces under standardized in vivo conditions.

Additional support comes from studies that have examined the effect of STL mesh resolution and mesh processing. Abad-Coronel et al. showed that exported digital models differed according to mesh resolution settings, especially in the number of mesh elements, although geometric differences among the resulting models were clinically small [14]. Similarly, Elbashti et al. demonstrated that triangular mesh reduction can affect geometric trueness when mesh reduction becomes substantial [15]. These studies are useful for the interpretation of the present results because they confirm that mesh structure is a meaningful characteristic of a digital model rather than a neutral technical detail. At the same time, these studies support an important nuance: a denser mesh does not automatically imply a clinically meaningful geometric advantage. This is particularly relevant in the present study, where V/A and F/A were deliberately interpreted as descriptors of local mesh representation rather than as direct indicators of superior or inferior scanning accuracy [14,15].

The present study also relates indirectly to the broader literature showing that digital outcomes depend not only on the scanner itself, but also on acquisition strategy. Hardan et al. showed in a systematic review and meta-analysis that scanning strategy affects the accuracy of complete-arch intraoral scans. This finding is important for the present work because it supports the decision to standardize the scanning workflow rigorously. All scans in the present study were acquired with the same scanner, by the same operator, using the same scanning protocol and dry clinical conditions. This methodological consistency strengthens the internal validity of the findings and reduces the likelihood that the observed inter-tooth differences were driven primarily by inconsistent scanning technique rather than by morphology-related variation [9].

Another important strength of the present study is its within-subject design. Because all investigated tooth types were analyzed within the same individuals, intersubject biological variability was reduced. This design improves sensitivity to localized morphology-related differences and represents an important methodological advantage over between-subject comparisons. In addition, all scans were acquired using the same intraoral scanner, by the same operator, and according to the same standardized scanning protocol, which reduced procedural variability. ROI selection was performed by a single trained examiner using predefined anatomical boundaries, while mesh analysis was conducted independently by a third examiner. Furthermore, predefined quality-control criteria were applied, and scans showing non-anatomical mesh defects affecting the intended ROI surfaces were excluded and replaced before final analysis. No mesh cleaning, smoothing, remeshing, or additional digital modification was applied after ROI export. Together, these methodological features support the interpretation that the observed differences more likely reflect true morphology-related variation in digital surface reconstruction rather than artifacts introduced by inconsistent acquisition, segmentation, or post-processing.

The novelty of the present study lies in the localized in vivo assessment of local mesh density distribution at the tooth level using the surface-normalized parameters V/A and F/A. Unlike previous studies that mainly focused on prepared teeth, whole-arch digital casts, export resolution, mesh reduction, or deviation-based accuracy outcomes, the present investigation compared multiple intact maxillary tooth types within the same individuals under a standardized clinical scanning workflow. This within-subject design allowed morphology-related variation in local mesh density distribution to be assessed while minimizing intersubject biological variability.

At the same time, several limitations should be acknowledged. First, the findings are specific to one scanner and one software ecosystem, namely the Medit i900 wired workflow, and cannot be generalized directly to other intraoral scanners or reconstruction algorithms. Because scanner hardware, software architecture, and proprietary reconstruction strategies may influence local mesh generation differently, the external validity of the present findings remains limited to this specific system. Validation across multiple intraoral scanner systems and, ideally, in multicenter settings would therefore be an important next step to determine whether the observed morphology-related differences in local mesh density are reproducible under different technological and clinical conditions. Second, only selected maxillary right teeth were included, and only their buccal surfaces were analyzed. Third, the sample consisted of a relatively homogeneous student population, which likely improved internal consistency but limited broader generalizability. Fourth, ROI selection remained examiner-dependent despite prior training and preliminary repeatability assessment, which included a comparison between two examiners on a small additional subset; the examiner with lower repeated-measurement variability was then selected for ROI delineation in the main study sample. Fifth, automatic hole closing formed part of the standardized workflow, although only when the ROI teeth themselves showed no defects, and this still represents a software-dependent component of processing. Finally, because the study was conducted in vivo and was not designed as a trueness study, the results should not be interpreted as indicating that one tooth type is clinically “better scanned” than another. Rather, the study indicates that different morphologies may be represented differently at the local mesh representation level [9,10,11].

The clinical and technical relevance of these findings should therefore be interpreted with caution. The present results do not imply that one tooth type is inherently more accurate in a conventional scanning sense. Instead, they suggest that different morphologies may generate different local mesh densities in STL reconstruction, even when the same scanner, operator, and scanning protocol are used. This may be clinically relevant because local differences in mesh density could influence how surface detail, contour transitions, and anatomically complex areas are represented in digital models used for downstream applications. In CAD/CAM workflows, this may affect the digital representation of tooth morphology during virtual design, particularly in regions where accurate surface detail is important for contour definition. In prosthetic workflows, differences in local digital surface representation may be relevant when restoration design depends on faithful reproduction of local surface form, even if the present study did not assess restoration fit directly. In digital orthodontics, variations in local digital surface representation may also influence visualization, segmentation, and digital analysis of tooth surfaces used for treatment planning and appliance design. However, because the present study did not evaluate manufacturing accuracy, restoration fit, or orthodontic outcomes directly, these implications should be interpreted as potential clinical relevance rather than as demonstrated clinical effects.

Further research should examine local mesh density distribution across additional tooth types, other anatomical regions, and multiple intraoral scanner systems, ideally within multicenter study designs. It would also be useful to investigate how export settings, software processing choices, and scanner-specific reconstruction algorithms influence local mesh density distribution and STL mesh representation. In addition, future studies should specifically investigate the relationship between local mesh density parameters and conventional accuracy outcomes, including trueness and precision, using study designs that allow direct comparison within the same surfaces and datasets. Such analyses were beyond the scope of the present study, which was not designed as a deviation-based accuracy study and did not include a reference dataset suitable for direct trueness assessment. Nevertheless, exploring whether V/A and F/A correlate with conventional accuracy metrics would be valuable for clarifying the interpretative significance of local mesh density findings under different acquisition and processing conditions.

In summary, the present study supports the view that tooth morphology influences local mesh density distribution in intraoral scanner-derived models. Under standardized in vivo scanning conditions, different maxillary tooth types exhibited significantly different surface-normalized mesh density values, suggesting that local anatomy affects the way digital tooth surfaces are reconstructed.

## 5. Conclusions

Within the limitations of this prospective within-subject observational study, the following conclusions were drawn:

1. Tooth morphology significantly influenced local mesh density distribution in intraoral scanner-derived STL models of selected maxillary teeth.
2. The surface-normalized mesh density parameters V/A and F/A differed significantly among the investigated tooth types, with the lowest values observed for the central incisor, intermediate values observed for the canine, and the highest values observed for the premolar and molar.
3. These findings suggest that local anatomical form affects the way tooth surfaces are digitally reconstructed into STL meshes under standardized in vivo scanning conditions, supporting local mesh density analysis as a useful complementary approach to conventional deviation-based digital assessments.

## Figures and Tables

**Table 1:** Inclusion and exclusion criteria.

Inclusion Criteria	Exclusion Criteria
Voluntary participation and signed informed consent	Refusal to participate or failure to provide informed consent
Presence of natural teeth in the selected regions of interest	Missing teeth in the selected regions of interest
Selected teeth free of caries	Presence of carious lesions on the selected teeth
Selected teeth without restorations (fillings, crowns, veneers, or other prosthetic reconstructions)	Presence of restorations on the selected teeth
Clinically healthy gingival tissues surrounding the selected teeth	Gingival inflammation or other periodontal soft-tissue pathology in the examined regions
Preserved tooth morphology suitable for digital surface analysis	Fractured, severely worn, malformed, or otherwise altered teeth that could affect mesh analysis

**Table 2:** Surface-normalized mesh density parameters according to tooth type and overall repeated-measures ANOVA results.

Variable	11	13	15	16	F	*p*
V/A (vertices/mm²)	18.2 ± 1.9	19.8 ± 1.4	23.8 ± 1.7	22.9 ± 2.0	62.36	<0.001
F/A (faces/mm²)	34.3 ± 3.6	37.5 ± 2.7	44.4 ± 3.3	42.9 ± 3.8	54.79	<0.001

## Data Availability

The data presented in this study are not publicly available due to privacy and ethical restrictions. Anonymized data may be made available by the corresponding author upon reasonable request, in accordance with ethical approval and applicable data protection requirements.
